# The environmental and genetic determinants of chick telomere length in Tree Swallows (*Tachycineta bicolor*)

**DOI:** 10.1002/ece3.5386

**Published:** 2019-07-04

**Authors:** Amos Belmaker, Kelly K. Hallinger, Rebbeca A. Glynn, David W. Winkler, Mark F. Haussmann

**Affiliations:** ^1^ Department of Ecology and Evolutionary Biology Cornell University Ithaca New York; ^2^ Department of Biology Bucknell University Lewisburg Pennsylvania; ^3^ Present address: The Steinhardt Museum of Natural History Tel‐Aviv University Tel Aviv Israel; ^4^ Present address: Department of Ecology and Evolutionary Biology University of Arizona Tucson Arizona

**Keywords:** brood enlargement, heritability, stress, *Tachycineta bicolor*, telomere length, Tree Swallow

## Abstract

Conditions during early life can have dramatic effects on adult characteristics and fitness. However, we still know little about the mechanisms that mediate these relationships. Telomere shortening is one possibility. Telomeres are long sequences of DNA that protect the ends of chromosomes. They shorten naturally throughout an individual's life, and individuals with short telomeres tend to have poorer health and reduced survival. Given this connection between telomere length (TL) and fitness, natural selection should favor individuals that are able to retain longer telomeres for a greater portion of their lives. However, the ability of natural selection to act on TL depends on the extent to which genetic and environmental factors influence TL. In this study, we experimentally enlarged broods of Tree Swallows (*Tachycineta bicolor*) to test the effects of demanding early‐life conditions on TL, while simultaneously cross‐fostering chicks to estimate heritable genetic influences on TL. In addition, we estimated the effects of parental age and chick sex on chick TL. We found that TL is highly heritable in Tree Swallow chicks, and that the maternal genetic basis for TL is stronger than is the paternal genetic basis. In contrast, the experimental manipulation of brood size had only a weak effect on chick TL, suggesting that the role of environmental factors in influencing TL early in life is limited. There was no effect of chick sex or parental age on chick TL. While these results are consistent with those reported in some studies, they are in conflict with others. These disparate conclusions might be attributable to the inherent complexity of telomere dynamics playing out differently in different populations or to study‐specific variation in the age at which subjects were measured.

## 1 INTRODUCTION

Early development is a critical life‐history stage, and organisms that successfully navigate this period can enjoy high fitness later in life, while individuals that endure early‐life stress may suffer later fitness deficits (Lindström, 1999; Watson, Bolton, & Monaghan, 2015). Despite its importance, we know little about the mechanisms mediating the effects of early‐life conditions on subsequent performance (Monaghan & Haussmann, 2006).

One mechanism by which development can affect fitness is through telomere shortening (Heidinger et al., 2012). Telomeres are long, repetitive, noncoding sequences of DNA that cap and protect the ends of chromosomes (Blackburn, 2000). As chromosomes shorten with each replication, there is a danger that important genetic information will be lost (Levy, Allsopp, Futcher, Greider, & Harley, 1992). Telomeres protect coding and structural DNA from degradation by bearing the brunt of chromosome shortening, leaving interior DNA sequences intact (Levy et al., 1992). Telomeres also prevent the DNA repair mechanism from falsely identifying chromosome ends as double‐stranded breaks (Nugent et al., 1998). When telomere length (TL) shortens beyond a certain threshold, the cell becomes senescent, starting a cascade that can lead to cell death, reduced organ function, and death of the individual (Campisi, 2005). Because short telomeres trigger this deleterious cascade, they are associated with poor health (Bojesen, 2013) and lower survival (Haussmann & Marchetto, 2010; but see McLennan et al., 2017), and have been used as a proxy for low quality in many species (i.e., Le Vaillant et al., 2015; but see Bauch, Becker, & Verhulst, 2013). In addition to the per‐replication shortening of TL (Levy et al., 1992), stress (Epel et al., 2004) and oxidative damage (Saretzki & Von Zglinicki, 2002) can hasten this process. In fact, many aspects of physiology are either directly or indirectly connected to telomere attrition, making TL a complex trait (Gatbonton et al., 2006; Haussmann & Marchetto, 2010). TL measured early in life can be a better predictor of fitness than can TL measured in later life (Heidinger et al., 2012). Thus, studying the causes of variation in early‐life TL will help us understand how TL can mediate the effect of developmental conditions on later performance and fitness (Watson et al., 2015).

Early‐life stress (Geiger et al., 2012), an individual's sex (Foote et al., 2011; Nicky et al., 2017) and the age (Arbeev, Hunt, Kimura, Aviv, & Yashin, 2011), and TL (De Meyer & Eisenberg, 2015) of the parents have all been shown to contribute to variation in early‐life TL. However, the effect each one has on TL is not consistent across studies. For example, while the individual's sex seems to play a key role in telomere dynamics and inheritance, the direction of the effect varies (Barrett & Richardson, 2011; Broer et al., 2013; Nordfjäll, Svenson, Norrback, Adolfsson, & Roos, 2009; Reichert, Rojas, et al., 2015; Nicky et al., 2017). The same is the case with parental age and chick TL—while the relationship between paternal age and the TL of offspring is well established in humans, in animals there seems to be much more variation (Arbeev et al., 2011; Asghar, Bensch, Tarka, Hansson, & Hasselquist, 2015; Broer et al., 2013; De Meyer et al., 2007; Eisenberg, Hayes, & Kuzawa, 2012; Ferlin et al., 2013; Froy et al., 2017; Kimura et al., 2008; Nawrot, Staessen, Gardner, & Aviv, 2004; Olsson et al., 2011; Prescott, Du, Wong, Han, & De Vivo, 2012; Unryn, Cook, & Riabowol, 2005). It is possible that the lack of a clear pattern arises because most predictors have been studied in isolation. It is therefore important to simultaneously study as many predictors as possible to understand the relative role each one plays in TL variation.

Because of the association between TL and fitness, we might expect natural selection to favor individuals who are able to maintain longer telomeres throughout their lives, especially in harsh environments. However, the ability of natural selection to act on TL depends on the extent to which TL is heritable. Estimates of TL heritability (*h* ^2^) range from 0.30 to 1.28 (Atema et al., 2015; Dugdale Hannah & Richardson David, 2018; note that *h* ^2^ values greater than one are possible if *h* ^2^ is estimated by regressing the offspring trait value on that of a single parent and multiplying by two). The individual's sex seems also to play a role in telomere inheritance: In several cases, the correlation between parental TL and that of the offspring has been found to be stronger for one sex than the other (Gardner et al., 2014; Reichert, Rojas, et al., 2015). It has been suggested that a combination of genetic imprinting and heterogamy could cause this sex‐specific pattern (Reichert, Rojas, et al., 2015), but the evidence in favor of this hypothesis is mixed (Broer et al., 2013; Eisenberg, 2014). Indeed, the sex‐specific pattern of TL inheritance seems to depend on a blend of biological and methodological factors (Broer et al., 2013; Eisenberg, 2014; De Meyer & Eisenberg, 2015; Nawrot et al., 2004; Olsson et al., 2011).

In this study, we tried to determine what factors predict TL in nestling Tree Swallows (*Tachycineta bicolor*). We cross‐fostered nestlings to generate a range of genetic relationships between nestlings reared in the same environment, and we manipulated brood sizes to generate two developmental contexts experienced by these nestlings. Thus, we were able to see how early‐life TL is influenced by both heritable and environmental factors acting simultaneously. Lastly, we noted both the sex of each chick and the age of the parents to estimate their roles in determining early‐life TL. The brood enlargement can affect chick TL either through environmental stress or through an indirect effect on chick growth. For this reason, we also include chick growth as a predictor in our model. Because the patterns observed in the literature for the factors we are measuring vary widely between species, predicting the effects of each is hard. As this study is more exploratory in nature, we do not add a detailed list of predictions. Studies that estimate TL heritability in wild populations, while simultaneously manipulating the developmental environment, are rare (Voillemot et al., 2012). By looking at all of these factors in the same system, we hope to gain insight into how each one, through its effect on TL, relates to fitness later in life.

## 2 METHODS

### 2.1 Study system and manipulation

**Figure 1:**
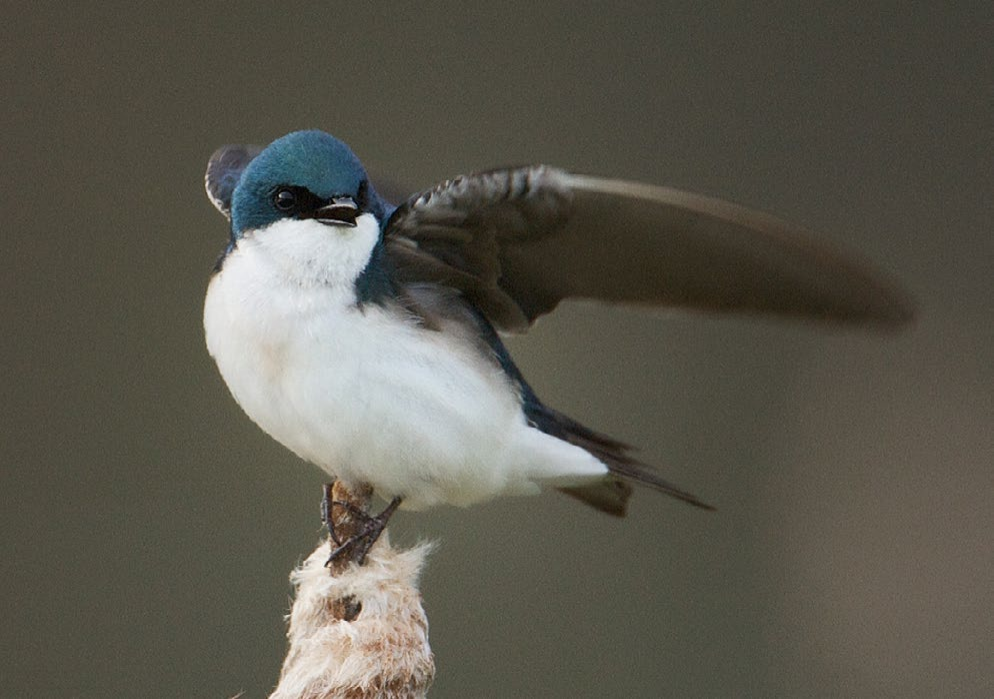
The Tree Swallow (*Tachycineta bicolor*)

The Tree Swallow (Figure 1) is a small, migratory, aerial insectivore that has been used as a model system for studies of traits ranging from life history and behavior to physiology (Jones, 2003). Telomere dynamics have been studied in Tree Swallows before (Haussmann, Winkler, Huntington, Nisbet, & Vleck, 2007; Haussmann et al., 2003; Haussmann Winkler, & Vleck, 2004, 2005; Ouyang, Lendvai, Moore, Bonier, & Haussmann, 2016) but no studies have examined the determinants of Tree Swallow TL in early life. The full details of the experimental manipulation have been published before (Belmaker, Hallinger, Glynn, Haussmann, & Winkler, 2018). However, for the benefit of the reader, and with permission from the authors, we fully describe the field, laboratory protocols, and the statistical methods used in detail.

During the breeding seasons of 2012–2014, we cross‐fostered and manipulated the brood sizes of Tree Swallows breeding in Harford, NY (42.44°N, 76.23°W). The study population bred in 130 man‐made nest boxes, which allowed us to closely monitor the breeding activity at the nest. Nests were monitored daily during laying to find the day the first egg was laid (“clutch initiation date”) in each occupied box, as well as the day the last egg was laid (“clutch completion date”). Females were caught in the box during mid‐incubation. Males were similarly captured during the nestling provisioning period. For each adult, we measured body mass, head‐plus‐bill length, and wing length. We also scored each individual's age using past banding records or plumage (Hussell, 1983). In cases where age was not precisely known (i.e., when the individual was in full adult plumage at the time of initial capture), we noted the minimum age of each individual (i.e., we assumed that the individual had been one [male] or two [female] years old during the first capture event; Hussell, 1983). Lastly, a blood sample was taken from the brachial vein for telomere length analysis and genotyping. Upon hatching, pairs of nests were matched for brood size, female age, and hatch date. We randomly selected one nest from each pair for brood enlargement and placed the second nest in the control treatment. We reciprocally transferred half of all nestlings between control and enlarged nests. This reciprocal transfer did not change the size of either brood but ensured that both pairs were raising both native and foreign young. We then added three nestlings to each enlarged brood, increasing its size by ~50% (Ardia, 2005). These additional nestlings were sourced from nests not assigned to either treatment, but whose nestlings had hatched at a similar time as the pair of experimental nests. We did not include a reduced‐brood treatment both to ensure the maximum possible sample size and because an artificially reduced brood might be interpreted by the adults as a partial‐predation event and skew their investment in unpredictable ways. Final brood sizes averaged 5.07 ± 0.67 and 8.05 ± 0.86 (mean ± *SD*) nestlings for control and enlarged broods, respectively. Following brood manipulation, we followed each breeding attempt to its conclusion.

Nestlings were individually marked by clipping toenails. For each chick, we measured mass, head‐plus‐bill, and wing length on days 0, 4, 8, and 12 posthatching. We halted chick measurements at the age of 12 days because after that the risk of premature fledging rises substantially. All morphometric measures were then combined into one size measure, using a principle component analysis. The first principle component was used as our size measure and explained 97% of the variance. All chicks that survived to day 12 were banded, and a blood sample was taken for telomere measurement and genotyping. A minimum of 20 and a maximum of 150 μl were taken into a heparinized microcapillary tube. Half of the blood was put into lysis buffer for genotyping and was stored at room temperature. The other half was put into an empty 1.5‐ml microcentrifuge tube and stored on ice until further processing in the lab. At the end of each day, telomere samples were spun down at 1098 rcf for 5 min, and the plasma was removed. One milliliter of NBS buffer (90% new‐born calf serum and 10% DMSO) was added and mixed with the red blood cells (RBCs). The samples were then frozen slowly and kept at −80°C for storage until analysis. Following banding, we continued to monitor nests to determine the date on which fledging occurred. After all surviving chicks had fledged, we noted the band number or marking of any dead chick left behind in the box. Any chicks that died before day 12 were genotyped, but because the TRF assay is sensitive to DNA degradation (Haussmann & Mauck, 2008), we did not estimate TL for these chicks.

### 2.2 Laboratory protocols

#### 2.2.1 Telomere length analysis

Telomere measurements were based on the TRF protocol described by Kimura et al. (2010). Samples were thawed at 37°C for 2 min and then spun down at 3,500 rpm for 5 min. The supernatant was discarded. DNA was extracted from the remaining RBCs using a Gentra Puregene extraction kit for the extraction of high‐quality, high‐yield DNA (Qiagen). In short, RBCs were lysed for at least an hour with proteinase K at 37°C. Proteins were precipitated out, and DNA was extracted using an isopropanol–ethanol extraction. DNA integrity was checked on a 0.8% agarose gel made with 1× TAE run for 1 hr in 120 V. Ten micrograms of DNA was digested for at least 16 hr at 37°C with a combination of three restriction enzymes (RsaI, HaeIII, and HinfI). Samples were then frozen until further processing. When ready for processing, samples were quickly thawed at 37°C and run on a 0.8% agarose gel in a pulsed‐field gel electrophoresis rig for 19 hr (3 V/cm, 0.5‐s initial switch time and 7‐s final switch time) along‐side three lanes of 1 kb extension ladder from Invitrogen and two standard lanes made of either Domestic Chicken blood or Tree Swallow blood. The gel was then dried and hybridized overnight with a radioactive probe (“CCCTAA” × 4) that anneals to the single‐stranded overhang at the end of the telomere. The next day the gel was washed with a 0.5× SSC solution and placed on a phosphor screen (Amersham Biosciences) for at least 2 days. The screen was then visualized using a Storm 540 Variable Mode Imager (Amersham Biosciences). It is important to note that the method we used does not denature DNA and so does not measure interstitial telomeric repeats that may skew our analysis (Nussey et al., 2014).

Because each chromosome may contain telomeres of different lengths, this procedure results in a smear rather than distinct bands of DNA. This smear represents the distribution of TLs *per individual* rather than one metric that summarizes that distribution (Kimura et al., 2010; Nussey et al., 2014). We quantified telomere distributions using ImageJ (version 2.0.0‐rc‐34/1.50a; Schindelin et al., 2012), an open‐source image processing software. Optical density values (OD) were measured along a line centered along each lane. Because one probe molecule attaches to one telomere molecule, the OD values directly correspond to the number of telomere molecules of the length indicated by the position on the gel. The fragment size of each telomere fragment at a given pixel location down the lane (KBi) was measured by fitting a cubic polynomial to the central ladder lane of each gel. We used an analysis window between 1.636 and 40 kb (the two outmost visible size markers). Background was subtracted from all OD measurements and was estimated by measuring a horizontal line placed just below the lowest size marker.

One of the advantages of using the TRF assay over other techniques is that each sample produces a distribution of TL *per individual* rather than one metric that summarizes that distribution (Nussey et al., 2014). This allows us to explore in greater depth how different characteristics of the TL distribution are involved in an individual's physiology. However, statistical methods that can analyze a distribution as one datum, both as a predictor and as a response, are new and still hard to implement (Ramsay, Hooker, & Graves, 2009). To balance the oversimplification of using only mean TL with the complex statistics involved in using the entire distribution, we measured the following key metrics from each distribution: the mean TL, the skew and kurtosis, and the tenth to ninetieth deciles of the TL distribution. With these metrics, we should have captured the main features of each distribution without overly complicating it. However, all these metrics were highly correlated (Table 1), and the implications of these correlations for TL measurement will be discussed in a different publication. Because all our metrics were correlated, we reduced the dimensionality of our TL measures with a principle component analysis (PCA) on all 12 metrics. This PCA was conducted using the “princomp” function from the “stat” package in R (version 3.2.1). We used only the first principal component score (PC1) for all analyses, as, by itself, it explained 88.5% of the variation. The loadings for PC1 are presented in the gray row in Table 1. Our measure of TL is this first component (PC1) where high PC1 scores represent longer mean TL and a distribution that is skewed toward longer telomere fragments. Low PC1 scores correspond to shorter mean TL and a short‐skewed fragment distribution. The coefficients of variation in our measurements of TL, based on standard samples run twice on each gel, are 9% and 5% for the two standards used, which is within the range reported in the literature. To further minimize the effects of gel ID on the results, we made sure to run paired broods on the same gel.

**Table 1:** The correlation coefficients (*r*) between 12 metrics from the TL distribution: mean TL, skew, kurtosis, and the 10th to 90th percentiles (P10 to P90, respectively)

	Mean	Skew	Kurtosis	P10	P20	P30	P40	P50	P60	P70	P80	P90
PC1 loadings	0.25	−0.08	−0.49	0.08	0.14	0.18	0.21	0.24	0.27	0.31	0.37	0.49
Mean	1											
Skew	−0.88	1										
Kurtosis	−0.84	0.95	1									
P10	0.72	−0.44	−0.33	1								
P20	0.87	−0.63	−0.52	0.95	1							
P30	0.92	−0.72	−0.61	0.88	0.99	1						
P40	0.95	−0.79	−0.68	0.83	0.96	0.99	1					
P50	0.97	−0.83	−0.74	0.78	0.93	0.98	0.99	1				
P60	0.98	−0.87	−0.79	0.74	0.89	0.95	0.98	0.99	1			
P70	0.99	−0.91	−0.84	0.68	0.85	0.91	0.95	0.97	0.99	1		
P80	0.98	−0.93	−0.9	0.61	0.77	0.84	0.89	0.93	0.96	0.99	1	
P90	0.92	−0.9	−0.94	0.46	0.63	0.71	0.76	0.81	0.86	0.9	0.96	1

Note

In all cases, *p* < 0.001. The gray row shows the PCA loadings for PC1, which explained 88.5% of the variation and was thus the only PC used in the analyses for this paper.

#### 2.2.2 Paternity analysis

To assign paternity, we extracted DNA from RBCs stored in lysis buffer or from dead nestlings using the QIAGEN DNeasy Blood and Tissue kit. Following extraction, we amplified nine microsatellite loci (Makarewich, Stenzler, Ferretti, Winkler, & Lovette, 2009; Stenzler, 2001) using multiplex polymerase chain reaction (PCR). PCR conditions are described fully in Belmaker et al. (2018). We used Geneious (version 9.0.5; Kearse et al., 2012) to call alleles and CERVUS (version 3.0; Kalinowski, Taper, & Marshall, 2007) to assign parentage to nestlings. We determined the sex of each nestling using a P2/P8 sexing protocol with a HaeIII digest similar to that described in Whittingham and Dunn (2000).

### 2.3 Statistical analysis

All analyses were carried out in R (version 3.3.2; R Core Team, 2015). We tested linear and generalized linear mixed‐effect models using the “lmer” and “glmer” functions from the “lme4” package (version 1.1‐11; Bates, Maechler, Bolker, & Walker, 2013).

#### 2.3.1 Treatment effect on chick size and mortality

To test the effects of the experimental manipulation on the size of chicks, we used a linear mixed‐effects model with the interaction of measurement number (out of the four total measurements taken) and experimental group as a fixed effect, and with chick “id,” natal, and rearing boxes as random effects. The probability of fledging was estimated using a generalized linear mixed model assuming a binomial distribution with experimental group as a fixed effect and natal and rearing boxes as random effects. *p*‐values for this analysis were obtained using a likelihood ration test calculated by the “ANOVA” function in R.

#### 2.3.2 The heritability of TL

We estimated the heritability of TL using a mid‐parent/offspring regression and estimated *h* ^2^ as the slope of this regression. For this analysis, we only used cases where we knew both genetic parents. We used the TL of each chick as our response variable and the average TL of its genetic parents as our predictor variable. We controlled for the effect of the experimental manipulation by including it as a predictor in the model. Natal box, rearing box, and the identity of the genetic father were added as random effects.

#### 2.3.3 The determinants of TL (Main model)

We estimated the effects of experimental group (fixed effect with two levels), parental TL (continuous covariate), and age (continuous covariate) and chick sex (fixed effect with two levels) on chick TL with a linear mixed‐effects model. We included parental TL and experimental group as fixed effects in order to test the influence of additive genetic and environmental factors, respectively, on nestling TL. To test growth per se as a mechanism by which the treatment could affect TL, we added nestling size at 12 days of age as a fixed effect (continuous covariate). We also added each chick's mass rank (continuous covariate), as it might be a better predictor of chick TL than growth (Nettle et al., 2015). To test whether parental age predicts chick TL, we added the minimum age of both genetic parents into the full model (continuous covariate). Lastly, to test whether parental and treatment effects vary with the sex of the chick, we included an interaction term with sex for each variable. To account for the fact that each adult parented several chicks and that chicks hatched or reared in the same box can be correlated, we added natal and rearing boxes as random effects. In addition, we added the ID of the genetic father as a random effect. We checked the variance inflation factor (VIF) for each of our factors, and none showed a sign of multicollinearity. We included only nestlings whose genetic parentage was known. Young of unknown paternity were excluded from all analyses (71% of all chicks).

To simplify this full model, we calculated the AICc (Akaike information criterion corrected for small‐sample sizes) for all possible combinations of the above‐described fixed effects using the “dredge” function in the MuMIn package in R (version 1.42.1; Barton, 2009). We then averaged all the models that were within a ∆AICc value of 2 from the model with the lowest AICc (models that are all equally likely as the “best” model) using the “model.avg” function in the MuMIn package. We then discuss the relative importance of each of our fixed effects after the model averaging process.

## 3 RESULTS

**Figure 2:**
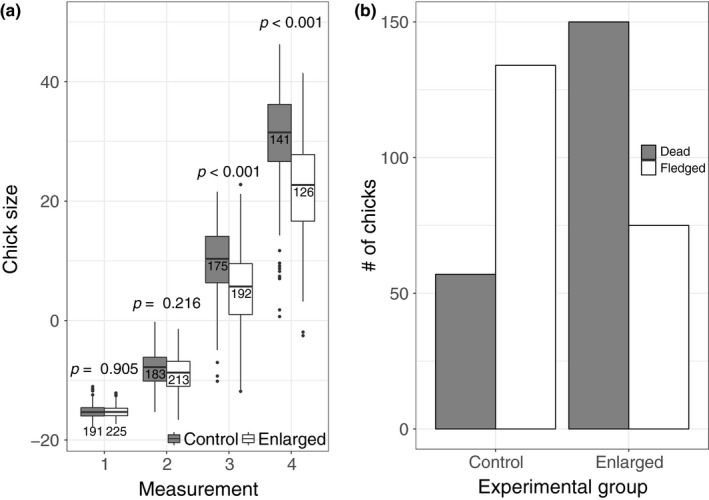
The effect of the brood enlargement on chick size (a) and the probability of fledging (b). Panel a shows a boxplot of the change in size with each measurement (out of four taken) in control (dark boxes) and enlarged (light boxes) broods. Dots are outliers and were calculated as ±1.5 × IQR, where IQR is the interquartile range. The numbers above each box are the sample size, and the *p*‐value (calculated from a linear mixed model) for the effect within each measurement is shown above the sample sizes. Panel b shows the number of chicks that fledged (light bars) or died (dark bars) in either control (left side) or enlarged (right side) broods. All chicks, including ones that died before the age of 12 days, are shown

In total, 39 paired‐brood manipulations were conducted (16 in 2012, 9 in 2013, and 14 in 2014) and 416 chicks were included in the experiment. At the start of the experiment, when the chicks were between 0 and 2 days old, there were no detectable size differences between chicks from enlarged and control broods (control chick size [PC1]: *n* = 191, −15.18 ± 1.14 [Mean ± *SD*]; size of chicks in enlarged broods [PC1]: *n* = 225, −15.29 ± 1.01; *t*_88.75_ = 0.12, *p* = 0.90; Figure 2a). Chicks growing up in enlarged broods grew more slowly than chicks in control broods, and size differences between them grew with each subsequent measurement (Figure 2a). In addition, chicks in enlarged broods were less likely to fledge (GLMM with binomial family: *n* = 416, *β* ± *SD* = −4.89 ± 1.27, , *p* < 0.001; Figures 2b).

**Figure 3:**
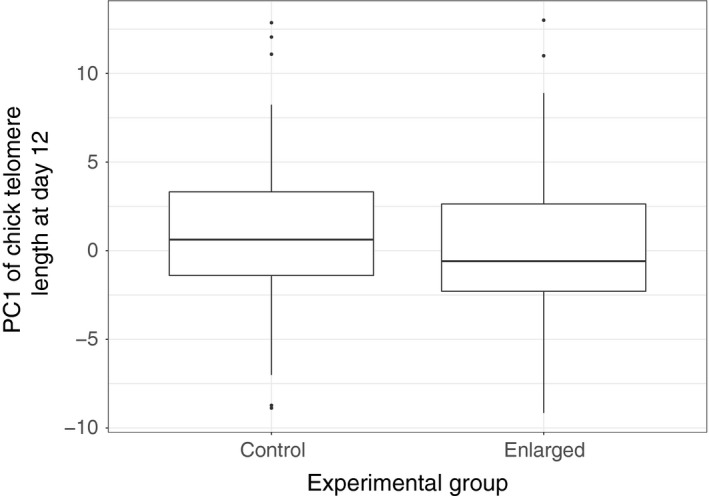
The effect of the brood enlargement on chick telomere length. Dots are outliers and were calculated as ±1.5 × IQR, where IQR is the interquartile range. Experimental group was not found to be an important predictor of chick telomere length based on AICc analysis

### 3.1 The heritability of TL

**Figure 4:**
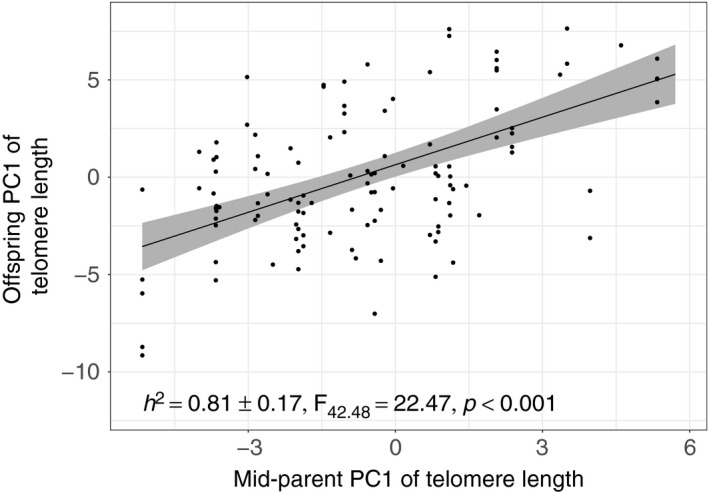
A plot of the correlation between the average telomere length of the parents and that of their offspring. The slope of the regression corresponds to the *h* ^2^ value and is estimated to be 0.81

The average TL of *genetic* parents was highly correlated with the TL of their siblings regardless of where those chicks were reared, and *h* ^2^ was estimated as 0.81 (LMM: *n* = 122, *β* = 0.81 ± 0.17, *F*_42.48_ = 22.47, *p* < 0.001; Figure 4). A brood‐level analysis that averaged TL across within‐pair offspring also produced a high estimate of *h* ^2^ (*n* = 37, *β* = 0.78 ± 0.19, *F*_35_ = 16.18, *p* < 0.001).

### 3.2 The determinants of TL (Main model)

**Figure 5:**
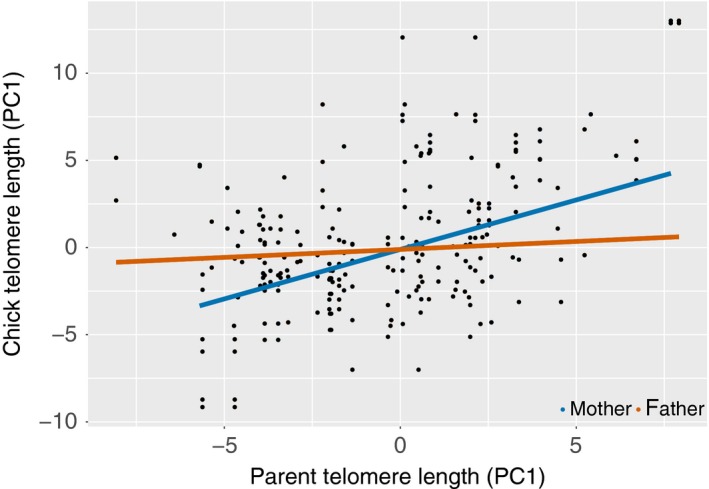
The relation between chick telomere lengths (TL) and those of their mothers (blue lines) and fathers (orange lines). While the telomere lengths of both mothers and fathers were found to be important predictors of chick TL, the correlation between mother and offspring is stronger than between father and offspring. The slope of the lines was drawn by averaging all relevant models that were within 2 AICc units from one another. Because the slope is an averaged estimate, we could not plot the confidence intervals

After removing missing values, sample size for all models in this analysis was 119 chicks in total. Fitting the set of all possible models shows 12 models to be within 2 AICc units of the best model (ΔAICc <2; Table 2). In the averaged model, maternal TL is the strongest predictor of chick TL, appearing in all 12 models and being the only predictor to have a statistically significant effect (Tables 3 and 4; Figure 5). The second most important predictor is chick size, but the effect size is small and nonsignificant (Tables 3 and 4). Paternal TL and experimental group have effects similar in importance, but neither is statistically significant (Tables 3 and 4; Figure 3). Maternal and paternal age and mass rank appear only in one or two models and are relatively unimportant in predicting chick TL (Table 4). The effects of chick sex and its interactions with other metrics were not strong enough to merit inclusion in any of the 12 top‐ranking models.

**Table 2:** The subset of 12 models that received the lowest AICc value from all possible models

Component factors	*df*	logLik	AICc	ΔAICc	Weight
Maternal TL Chick size at day 12	7	−298.25	611.5	0	0.14
Experimental group Paternal TL Maternal TL	8	−297.26	611.83	0.32	0.12
Paternal TL Maternal TL	7	−298.46	611.93	0.43	0.11
Paternal TL Maternal TL Chick size at day 12	8	−297.31	611.93	0.43	0.11
Experimental group Maternal TL	7	−298.73	612.47	0.97	0.08
Paternal age Maternal TL Chick size at day 12	8	−297.61	612.53	1.03	0.08
Maternal TL	6	−300.11	612.97	1.46	0.07
Experimental group Maternal TL Chick size at day 12	8	−297.83	612.97	1.46	0.07
Maternal TL Mass rank Chick size at day 12	8	−297.89	613.09	1.58	0.06
Experimental group Paternal age Maternal TL	8	−297.95	613.22	1.71	0.06
Experimental group Paternal TL Maternal TL Chick size at day 12	9	−296.82	613.3	1.79	0.06
Maternal age Maternal TL Chick size at day 12	8	−298.05	613.41	1.91	0.05

Note

All 12 models have ΔAICc <2 from the model with the lowest AICc and were subsequently averaged.

**Table 3:** The averaged coefficients for seven fixed effects from 12 models included in the analysis

	Estimate	*SE*	Adjusted *SE*	*Z* value	Pr(>|*z*|)
Full average					
Intercept	−0.11	1.43	1.44	0.08	0.94
Maternal TL	0.57	0.14	0.14	3.97	**<0.001**
Chick size	0.04	0.04	0.04	0.84	0.40
Experimental group	−0.34	0.60	0.60	0.57	0.57
Paternal TL	0.09	0.14	0.14	0.64	0.52
Paternal age	0.05	0.17	0.17	0.30	0.77
Mass rank	0.01	0.06	0.06	0.17	0.87
Maternal age	0.01	0.11	0.11	0.12	0.90
Conditional average					
Intercept	−0.11	1.43	1.44	0.08	0.94
Maternal TL	0.57	0.14	0.14	3.97	**<0.001**
Chick size	0.06	0.04	0.04	1.64	0.10
Experimental group	−0.91	0.66	0.67	1.35	0.18
Paternal TL	0.23	0.14	0.14	1.69	**0.09**
Paternal age	0.35	0.30	0.30	1.17	0.24
Mass rank	0.15	0.17	0.17	0.86	0.39
Maternal age	0.26	0.42	0.42	0.62	0.53

Note

Bolded rows are significant at the 10% level. Full averages mean that coefficients of zero are also included and conditional averages omit these. Here, we included both but they do not differ in any substantial way.

**Table 4:** The relative importance and the number of models that contained each of seven fixed effects for predicting Tree Swallow chick telomere length

	Relative variable importance						Maternal age
	Maternal TL	Chick size	Paternal TL	Experimental group	Paternal age	Mass rank	
Importance	1	0.57	0.39	0.38	0.14	0.06	0.05
*N* containing models	12	7	4	5	2	1	1

Note

These are based on an average model—of the list of all possible models, all the models with the lowest AICc score and within a range of 2 (∆AICc < 2) were averaged.

## 4 DISCUSSION

In this study, we evaluated the relative roles of genetics and environment in affecting Tree Swallow chick TL. Our results indicate a strong role for maternal inheritance and weak or no effects of brood enlargement, parental age, and chick sex or size on chick TL. The strong role for genetic effects on chick TL is indicated by both the high estimated *h* ^2^ value (0.81) and the fact that both maternal and paternal TL were among the most important predictors of chick TL in the full mixed model (Tables 3 and 4). Previous studies offer a wide range of estimates for TL heritability, with the measures that we obtained falling at the high end of this range (Atema et al., 2015; Dugdale Hannah & Richardson David, 2018). While these high heritability estimates may in part be due to maternal effects that are expressed prior to hatching, such as egg composition or incubation, the weak effect of the brood enlargement combined with the strong effect of parental TL in the main model suggest that, at least at the age of 12 days old, Tree Swallow TL is determined more by heritable genetic factors than by environmental ones.

How do these results compare with what is already known about early‐life TL? For each of the metrics we measured to explain chick TL, a wide range of patterns is observed in the literature: First, past experiments with group enlargements either succeed (Nettle et al., 2015) or fail (Reichert, Criscuolo, et al., 2015; Voillemot et al., 2012) to show an effect of the treatment on chick TL. Here, we find only a very weak effect of the brood enlargement and chick size. Second, even though a parental age effect on chick TL is often found, across species there seems to be variation in how parental age affects TL (Arbeev et al., 2011; Asghar et al., 2015; Broer et al., 2013; De Meyer et al., 2007; Eisenberg et al., 2012; Ferlin et al., 2013; Froy et al., 2017; Kimura et al., 2008; Nawrot et al., 2004; Olsson et al., 2011; Prescott et al., 2012; Unryn et al., 2005). Here, we find no effect of parental age at all. In our data, 48% of females and 68% of males were older than the average age of two, and we sampled individuals as old as five and six, respectively. This means the range of samples alone cannot explain the lack of effect of parental age we report. Lastly, the nature of the sex‐specific pattern of TL inheritance (whether male or female biased) varies across studies (Broer et al., 2013; Eisenberg, 2014; Nawrot et al., 2004; Olsson et al., 2011). In our study, we report that heritability is stronger through the female than the male. It seems that the effects of each of these factors on TL are study‐specific, so how should we make sense of these diverse patterns?

One potential solution is to remember that TL is a dynamic and complex trait (Gatbonton et al., 2006; Haussmann & Marchetto, 2010) that changes throughout an individual's life and is affected by both internal and external factors. This complexity, the many interconnected factors that affect TL, the variation in life history among species and between years, may make it difficult to predict the effect of any one factor on TL in a given system. This could be responsible for the study specificity we observe.

Alternatively, the timing of measurement of both adults and offspring can create many contrasting patterns. For example, in this study chicks were sampled once, at 12 days of age. It is possible that this affected the results in a couple of ways. First, as chicks that died before reaching this age were not sampled, possibly short‐telomere, low‐quality chicks that did not survive to be sampled were overrepresented among dead chicks (Heidinger et al., 2012), causing the effect of brood enlargement on TL to appear limited. Second, Tree Swallow chicks fledge closer to the age of 21 days rather than 12. Thus, chicks continued to experience the consequences of the brood manipulation long after we took our measurements. It is possible that effects on TL may only have become apparent after this time and, even though the chicks would have completed most of their growth by 12 days of age (Winkler & Adler, 1996). Had we measured the chicks closer to fledging, or even postfledging, it is possible that we would have observed a larger difference between the experimental groups. Twelve days of elevated competition during the most active phase of chick growth may (Nettle et al., 2015) or may not (Reichert, Criscuolo, et al., 2015; Voillemot et al., 2012) be enough to induce differences in TL, depending on the specifics of the study and species. So while harsh conditions *do* contribute to telomere shortening, the duration and intensity of the treatment needed to induce this shortening may vary. For Tree Swallows, it would seem that 12 days of a brood enlargement is not sufficient to induce much variation in TL.

Another explanation for the high degree of variation in TL effects across studies is a potential interaction between parental TL effects (heritability) and parental age effects. Before development starts, the zygote inherits its telomeres from the gametes of the parents (Graakjaer et al., 2004; De Meyer et al., 2014). The TL of the *specific* sperm and egg forming the zygote will determine its TL—a parental TL effect on chick TL. Any age‐specific process that affects sperm and egg TL will influence the pool from which gamete telomeres can be chosen and will consequently affect offspring TL—an age effect on chick TL. Age‐related telomere shortening (Hall et al., 2004; Haussmann & Marchetto, 2010; Haussmann et al., 2003), telomerase activity in the germ line of adult birds (Haussmann, Winkler, Huntington, Nisbet, & Vleck, 2004; Haussmann et al., 2007), a reduction in sperm quality with age (Ferlin et al., 2013; Rocca et al., 2016; Waeleghem, Clercq, Vermeulen, Schoonjans, & Comhaire, 1996), TL‐based selective stem cell turnover (Kimura et al., 2008), and stochastic processes during sperm maturation that increase variability in sperm TL as the individual ages (De Meyer & Eisenberg, 2015) could all increase the variability of TL in the gametes from which the zygote is formed (De Meyer & Eisenberg, 2015). Small‐sample random sampling from this distribution could produce many possible patterns of parental age effects.

The above discussion has important implications for the way we view the ability of natural selection to act on TL variation. On the one hand, TL is inherited directly from the gametes of the parents (Graakjaer et al., 2004; De Meyer et al., 2014), but throughout the subsequent life of the zygote, decreases in its TL can be countered by TL maintenance mechanisms that are also inherited from the parents (Hjelmborg et al., 2015). These mechanisms could be anything from systems that deal with environmental stress to ones aiding in foraging. Indeed, telomere shortening *rate* has been shown to affect fitness irrespective of telomere *length* (Bize, Criscuolo, Metcalfe, Nasir, & Monaghan, 2009; Epel et al., 2009; Salomons et al., 2009), and shortening rate has been shown to be heritable as well (Hjelmborg et al., 2015). Both heritable variation in the base telomere sequence and the telomere‐maintenance mechanisms can help produce correlations between parents and offspring. If we were to measure TL in chicks soon after hatching, the influence of the base telomere sequence inherited from the parents would dominate any inherited similarity based on shared telomere‐maintenance genes (De Meyer et al., 2014). In contrast, if chicks are measured when they are older, the environment will have had a chance to decrease the chick's TL, and inborn repair mechanisms can act on any such erosion. Thus, as chicks age, the stochastic nature of environmental challenges, together with genetic variation in the effectiveness of repair mechanisms, can present many avenues to reduce the similarity between parents and offspring. A study in King Penguins (*Aptenodytes patagonicus*) shows this exact pattern: TL was found to be maternally inherited when the chicks were 10 days old, but there was no significant heritability at older chick ages (Reichert, Rojas, et al., 2015). TL is clearly a dynamic character. When we compare the TL of parents and offspring, we are comparing measures at two very different life stages where the relative importance and histories of environmental stressors and inherited influences may differ. Because both the initial telomere sequence and the mechanisms of telomere repair are inherited, a correlation between parents and offspring might be expected at any combination of their relative ages, but that underlying similarity may arise through very different pathways.

It is important to bear this in mind when considering natural selection's ability to shape TL variation. Inheritance of a long‐telomere base sequence can give an individual an early advantage, but without a good mechanism to maintain those long telomeres, an individual will suffer the deleterious effects of telomere erosion. An individual born with short telomeres but with an efficient telomere‐maintenance system can still benefit greatly from keeping its telomeres from shortening further. When we try to estimate natural selection's ability to mold TL variation, we need to keep in mind that, depending on the life stage in which we are measuring heritability, we could be measuring the heritability of very different things.

## CONFLICT OF INTEREST

All authors declare no conflict of interest.

## AUTHORS’ CONTRIBUTIONS

AB and DWW conceived the ideas and designed methodology. AB, RAG, and KKH collected the data. AB and KHH analyzed the data. AB, KHH, MFH, and DWW led the writing of the manuscript. All authors gave final approval for publication.

## ETHICAL APPROVAL

Data collection was done under federal collecting permit (M8757670‐0) given to David Winkler. All procedures performed in this study were in accordance with the ethical standards of Cornell University.

## Data Availability

The data associated with the publication have been uploaded to Dryad (https://datadryad.org/) and have been assigned the https://doi.org/10.5061/dryad.vb658bc.
